# Single Amino Acid Substitution in the DNA Repairing Gene *Radiation-Sensitive 4* Contributes to Ultraviolet Tolerance of a Plant Pathogen

**DOI:** 10.3389/fmicb.2022.927139

**Published:** 2022-07-14

**Authors:** Yan-Ping Wang, Li-Na Yang, Yuan-Yuan Feng, Songqing Liu, Jiasui Zhan

**Affiliations:** ^1^Sichuan Provincial Key Laboratory for Development and Utilization of Characteristic Horticultural Biological Resources, College of Chemistry and Life Sciences, Chengdu Normal University, Chengdu, China; ^2^Institute of Oceanography, Minjiang University, Fuzhou, China; ^3^Department of Forest Mycology and Plant Pathology, Swedish University of Agricultural Sciences, Uppsala, Sweden

**Keywords:** nucleotide excision repair system, climate change, UV adaptation, population genetics, natural selection, transcriptional regulation, evolutionary ecology, agriculture

## Abstract

To successfully survive and reproduce, all species constantly modify the structure and expression of their genomes to cope with changing environmental conditions including ultraviolet (UV) radiation. Thus, knowledge of species adaptation to environmental changes is a central theme of evolutionary studies which could have important implication for disease management and social-ecological sustainability in the future but is generally insufficient. Here, we investigated the evolution of UV adaptation in organisms by population genetic analysis of sequence structure, physiochemistry, transcription, and fitness variation in the *radiation-sensitive 4* (*RAD4*) gene of the Irish potato famine pathogen *Phytophthora infestans* sampled from various altitudes. We found that *RAD4* is a key gene determining the resistance of the pathogen to UV stress as indicated by strong phenotype-genotype-geography associations and upregulated transcription after UV exposure. We also found conserved evolution in the *RAD4* gene. Only five nucleotide haplotypes corresponding to three protein isoforms generated by point mutations were detected in the 140 sequences analyzed and the mutations were constrained to the N-terminal domain of the protein. Physiochemical changes associated with non-synonymous mutations generate severe fitness penalty to mutants, which are purged out by natural selection, leading to the conserved evolution observed in the gene.

## Introduction

The unprecedented climate change, believed to be mainly caused by improper anthropocentric activities (Fawzy et al., 2020; Manzanedo and Manning, 2020), is one of the main challenges the contemporary society is facing (Harmer et al., 2020). As an important component of climate change, increasing ultraviolet (UV) radiation on the Earth’s surface can greatly threaten human, animal, and environmental health, either directly or indirectly, by affecting the disease emergence and epidemics (Hill et al., 2011), food production, landscape structure, and ecological functions (Hisano et al., 2018). Elevating UV radiation occurs as a result of stratospheric ozone layer depletion due to excess releases of industrial and inhabitant pollutants such as chlorofluorocarbons, chlorocarbons, and organo-bromides to the atmosphere (Shindell et al., 1998). Despite the progressive ban in using these hazardous substances in the world, the level of UV radiation on the Earth remains of great concerned (Watanabe et al., 2011; Araújo et al., 2021). UV is a harmful substance causing deleterious effects on all living organisms ranging from prokaryotic bacteria to eukaryotic organisms including lower and higher plants, animals, and humans (Sinha and Häder, 2002). In addition, UV can stimulate the release of volatile organic compounds from soil and plants, increasing greenhouse gases in the atmosphere and hence global warming (Bornman et al., 2015).

Genotoxicity is the main harm of UV lights to organisms. It damages fundamental biomolecules of cells, leading to genomic instability or modification (Tabazadeh et al., 2000), and consequently influencing genetic, biochemical, morphological, physiological, and ecological processes of species (Tyrrell, 1984; Blume et al., 2012). Over the period of evolutionary adaptation, organisms have developed an array of genetic and physiological mechanisms to protect against UV damages (Franks and Hoffmann, 2012; Braga et al., 2015). For example, plants can reduce UV damage by adjusting their leaf sizes and shapes (e.g., curling leaves and shiny wax coating) as well as petiole angle (Nedunchezhian and Kulandaivelu, 1996), or upregulating the production of antioxidative compounds (Wargent and Jordan, 2013). When exposed to UV stress, some pathogens can invade hosts to escape UV damages (Rasanayagam et al., 1995) or produce specific compounds such as melanin, carotenoid, lignin, and mycosporine-like amino acid to block the radiation effects (Ruisi et al., 2006).

Species adaptation to UV radiation can also be achieved by repairing system after damage occurs. Because DNA is one of the key targets of UV-induced damage that generates a permanent and irreversible consequence on species prosperity, DNA repairing exists almost in all species stretching from lower organisms of bacteria (Fusco et al., 2020) and cyanobacteria (Pathak et al., 2019) to higher organisms of macroalgae, plants (Buma et al., 2001), animals, and humans (Sinha and Häder, 2002). When DNA is irradiated by UV lights, energy from the photon induces the formation of covalent linkages between adjacent pyrimidine bases, generating cyclobutane pyrimidine dimers (CPDs), and pyrimidine 6-4 pyrimidone photoproducts (6-4 PPs) to break DNA stands (Rastogi et al., 2010). If unrepaired, the broken stands inhibit DNA replication and transcription, which in turn, not only retard cell growth and development (Hollósy, 2002), increase apoptosis (Ryu et al., 2019), but also may result in mutations (Lahari et al., 2018), and further development of various diseases in human (Sivamani et al., 2009). In pathogens, cumulative UV damage adversely influences their pathogenicity (Milo-Cochavi et al., 2019).

Species have developed both light-dependent (photoactivation) and light-independent (Nucleotide Excision Repair, NER) mechanisms to repair UV-damaged DNA (Sancar, 2016). As an evolutionarily conserved but biologically versatile system, NER recruits many proteins and other molecules to entail a series of biochemical activities for initiating damage recognition, assembling repair protein complexes, unwinding DNA double helix, excising the damage and synthesizing DNA fragment using the intact strand as a template to fill the gap, and ligating (Rastogi et al., 2010). Among them, radiation-sensitive 4 (RAD4) is a key protein regulating NER activities in the cells (Min and Pavletich, 2007). It interacts with RAD23, another radiation-sensitive protein, to form a heterodimer (NEF2) and initiate damage recognition and recruitment of other proteins required for NER functions (Guzder et al., 1998). The RAD4 protein contains an N-terminal including the transglutaminase-homology domain (TGD) and three beta-hairpin domains (BHD1, BHD2, and BHD3). TGD and BHD1 bind to a segment of undamaged, double-strand DNA, whereas BHD2 and BHD3 bind to the UV-damaged DNA lesion (Min and Pavletich, 2007). The four domains work together to open up the damaged DNA by inserting a β-hairpin into the duplex and flip out the damage (Velmurugu et al., 2016).

*Phytophthora infestans* (Mont) de Bary, a pathogenic oomycete in *Straminipila* group (Klinter et al., 2019), is a model organism in plant pathology due to its tremendous effect on human history. The resultant potato late blight disease triggered the Irish potato famine (Fry and Mizubuti, 1998), leading to human catastrophe of millions of starvation, death, and migration, and remains as one of the main threats to potato production and food security worldwide due to the remarkable adaptation ability of the pathogen to control strategies and climate change (McDonald and Linde, 2002; Wu et al., 2020). The high evolvability of the pathogen is likely attributed to its large genome size enriched with transposable elements (Fouché et al., 2022), complex inheritable nature (Win et al., 2006), and multiple reproduction systems (Reedy et al., 2009). UV is an important factor that regulates the life cycle and infection process of *P. infestans*. Elevated UV radiation associated with climate change is expected to have a considerable impact on the future epidemics of the pathogen (Fustier et al., 2019). Our previous studies revealed significant intra- and inter-population variations in UV tolerance of *P. infestans.* Although phenotypic plasticity resulted from gene and pathway regulation is the main determinant of UV tolerance in the pathogen (Wu et al., 2019), genetic modification in key DNA-repairing genes also contributed to the adaptation. Spatial heterogeneity in *RAD23* of *P. infestans* is tightly linked to both geographic variation of UV radiation in fields and genetic variation of UV tolerance in the pathogen (Wang et al., 2021). The study also revealed that *RAD23* is highly conserved with a limited source of genetic variation in the pathogen and many other species (Bertolaet et al., 2001). However, studying evolutionary mechanisms and contribution of *RAD4* to UV adaptation in plant pathogens is rare but important in effectively preventing and controlling plant diseases in the face of increasing future UV radiation.

Therefore, the specific objectives of this study were to (1) determine the role of *RAD4* in UV adaptation of *P. infestans*; (2) explore the genetic variation and spatial distribution of the *RAD4* gene; and (3) decode the genetic mechanisms governing the evolution of *RAD4*. To achieve these objectives, we compared sequence characteristics of 140 *RAD4* genes generated from seven populations of *P. infestans* isolates. Transcriptional regulation of the gene, biochemical features of deduced proteins, and UV adaptation of the pathogen isolates were analyzed and compared. We found low genetic variation but spatial polymorphism in *RAD4*. Both the genetic structure and expression regulation of *RAD4* contributed to the UV adaptation of *P. infestans*.

## Materials and Methods

### *Phytophthora infestans* Collections

During 2010 and 2011, diseased potato leaves carrying single *P. infestans* lesion were sampled from seven potato fields located in Guangxi, Guizhou, Ningxia, Fujian (Fuzhou and Ningde), Yunnan, and Gansu (Qin et al., 2016; Wu et al., 2016; Yang et al., 2016). The several locations separated by 141–1,756 km geographically differ largely in ecological niches, landscape structure, and cropping systems such as altitude (Supplementary Table 1), temperature, *P. infestans* epidemics, and potato production season and size (Wang et al., 2021). In each of the seven collections, infected leaves were randomly sampled from plants grown in the same field but separated by >100 cm from each other and then transported to the laboratory. For pathogen isolation, running tap water followed by sterilized distilled water was used to clean mud on the infected leaves. Some mycelia on the edge of sporulation lesions were picked by a sterile inoculating needle and transferred into a rye B agar plate supplemented with ampicillin (100 μg/ml) and rifampicin (10 μg/ml). The plates were kept at 19°C in the dark for 7 days to develop colonies. The isolations were purified at least two times by transferring mycelium from the resulted colony to a fresh rye B plate. Restriction enzyme polymerase chain reaction (PCR) amplification of mitochondrial haplotypes, SSR assay of nuclear genomes, mating type determination, and sequence analysis of a housekeeping gene and effector gene were applied to determine the genotypes of these isolates (Knapova and Gisi, 2002; Flier et al., 2003; Lees et al., 2006). A total of 140 distinct genotypes with 20 from each of the seven field populations were selected for the study. Details of *P. infestans* collection, isolation, purification, and molecular characterization of these isolates were described in the previous publications (Qin et al., 2016; Yang et al., 2016; Wu et al., 2020; Lurwanu et al., 2021).

### *RAD4* Sequence Generation and Analysis

#### Genomic DNA Extraction and *RAD4* Sequence

To extract genomic DNA, the 140 isolates retrieved from a long-term storage were cultured on rye B agar supplemented with ampicillin (100 μg/ml) and rifampin (10 μg/ml) in the dark at 19°C for 15 days. Mycelia of the isolates were harvested, transferred into a sterile centrifuge tube (2 ml), and lyophilized using a vacuum freeze dryer (Alpha1-2, Christ). Genomic DNAs of the isolates were extracted from the lyophilized mycelia with gDNA kit (Promega Biotech. Co. TRANSGEN, Beijing, China) according to the manufacturer’s instructions.

Full *RAD4* gene was amplified by three pairs of specific primers (Supplementary Table 2), which were designed from the reference sequence (NCBI number 9474425). PCR amplifications were performed in a reaction mixture containing 1.0 μl of template DNA, 1.0 μl of each primer (10 μmol/L), 13 μl of 2 × EasyTaq^®^ PCR SuperMix (+dye) (TransGen Biotech, Beijing, China), and nuclease-free water in a final volume of 25 μl. The PCR program was started by 94°C DNA denaturation step for 5 min, followed by 35 cycles of amplification for 30 s at 94°C, 30 s annealing at special time basing on primer, and extension at 72°C, and ended with a further extension step at 72°C for 10 min. PCR products were separated by gel electrophoresis (1%), ligated into T5 zero cloning vector after purification and then transformed with Trans1-T1 into competent cells by a heat-shock process at 42°C for 30 s (pEASY^®^-T5 Zero Cloning Kit). A total of four colonies were randomly picked from each transformation and incubated on Luria-Bertani liquid media at 37°C for 16 h under continuous shaking. One colony was randomly picked and sequenced by Sangon Biotech Co., Ltd. (Sangon, China) using an ABI3730XL DNA Analyzer.

#### Sequence Alignment and Genetic Variation Estimate

Sequence peak was visualized by Chromas^1^ and manually edited to remove the potential fake “mutations” caused by PCR artifacts (Yang et al., 2013). The complete sequence was constructed by assembling three partial sequences generated from different primers using Fragment Merger (Bell and Kramvis, 2013). Multiple sequence alignment was performed using ClustalW embedded in MEGA 7.0.21 (Sudhir et al., 2016). Nucleotide haplotypes were constructed with the PHASRE algorithm implemented in DnaSP 6 (Rozas et al., 2017). Nucleotide composition in the *RAD4* sequences was estimated using BioEdit Sequence Alignment Editor (Hall, 1999). Homogeneity of nucleotide proportions in the *RAD4* sequences, haplotype frequency, and isoform frequency among the populations were evaluated by λ^2^ tests (Kathleen et al., 2017). A median joining (MJ) haplotype network was generated by DanSP6 for nucleotide sequences and visualized by PopART 1.7 (Leigh and Bryant, 2015).

### Biophysical and Biochemical Analysis of RAD4 Protein

Amino acid isoforms were deduced by MEGA 7.0.21 and displayed by the online program ESPript.^2^ The deduced isoforms were coded with “Iso” followed by a number and analyzed for amino acid compositions by BioEdit Sequence Alignment Editor. The RePROF algorithm embedded in the online tool PredictProtein^3^ was used to construct and annotate the secondary structure and solvent accessibility of RAD4 proteins based on multiple sequence alignments (MSAs) and multi-level systems. Hydrophobicity was evaluated by the Kyte & Doolittle (K-D) approach (Kyte and Doolittle, 1982) embedded in BioEdit Sequence Alignment Editor. IUPred2A^4^ was used to predict functional domains and their positions of RAD4 protein. The intrinsically disordered protein regions (IDPRs) were predicted by the online tool MobiDB.^5^

### Ultraviolet Treatment and Quantitative Real-Time PCR Analysis

A total of four Iso-1 *Phytophthora infestans* isolates were selected from the collections and exposed to UVC for 24 min using the ultraviolet light C lamp (PHILIPS, wavelength = 254 nm, 30 w) placed 50 cm above the colony. The UV exposure was repeated every 24 h for 8 days. On the 9th day, ∼150 mg mycelia of the UV-treated isolates and controls (without UV treatment) were transferred into cryopreservation tubes and frozen by liquid nitrogen. The frozen mycelia were ground to powder by mortar and RNA were extracted using a TransZol Up Plus RNA Kit following the manufacturer’s instructions (Transgen, Beijing, China). The concentration and quality of RNA were determined by a NanoDrop spectrophotometer (Thermo Fisher Scientific, Inc., Shanghai, China) using an absorbance ratio of OD260/280. First-strand cDNA was synthesized using 1 μg total RNA, Anchored Oligo (dT)_18_ primers by TransScript One-Step gDNA Removal and cDNA Synthesis SuperMix (Transgen) according to the manufacturer’s instructions. Synthesized cDNA was stored at –20°C refrigerator until use. The details of UV treatments were described in a previous publication (Wang et al., 2021).

The specific *RAD4* qRT-PCR primers (F: 5′- TGTTCAGCC ACTTCGGTCAGC-3′ and R: 5′-GTTGCCTCTTGCCTGCCA CT-3′) were designed by Premier 6.0 from the reference CDS sequence (PITG_10602). *Actin A* gene (NCBI, M59715) was used as the internal control (Avrova et al., 2003), and the 2^–△△*CT*^ method was used to calculate the relative expressions of *RAD4* to *Actin A* gene (Livak and Schmittgen, 2001). Quantitative real-time PCR (qRT-PCR) was performed on a QuantStudio™ 5 Real-Time PCR System. The 20 μl reaction system contained 2.0 μl of diluted cDNA, 10 μl of Hieff^®^ qPCR SYBR Green Master Mix (Yeasen Biotech Co., Ltd., Shanghai, China), 0.40 μl of 50 × Low ROX, 0.40 μl of each primer, and 6.80 μl RNase-free water. The cycling parameters were as follows: started at 95°C for 5 min, followed by 40 cycles of 95°C for 10 s, 60°C for 20 s, 95°C for 15 s, 60°C for 1 min, and 95°C for 1 s.

### Tolerance of *RAD4* Mutants to Ultraviolet Radiation

The 140 isolates were exposed to UVC light for 10, 15, 30, 90, 180, 300, and 480 s, respectively, using the ultraviolet light C lamp as described in Section Ultraviolet Treatment and Quantitative Real-Time PCR Analysis. Mycelial plugs (ϕ = 5 mm) from the margin of UVC exposed colonies were transferred to fresh rye B plates with three replicates and then kept in dark incubator at 19°C for 8 days. Control plates (without UVC treatment) were also included for each isolate. Colony sizes, starting from the 3rd day after inoculation until the 8th day after inoculation, were measured by the image analysis software ASSESS (Lamari, 2002). Colony growth rate was estimated using a logistic model (Aguayo et al., 2014) based on colony sizes measured during the experimental days for each of UV treatment time. UV tolerance was estimated by calculating the relative growth rate (RGR) of the isolates treated by UVC to the controls. RGR comparison among RAD4 isoforms was analyzed using ANOVA followed by Duncan’s multiple range test (Duncan, 1955) in SPSS (SPSS Inc. v.11.0).

## Results

### Genetic Variation in Nucleotide Sequence

**FIGURE 1:**
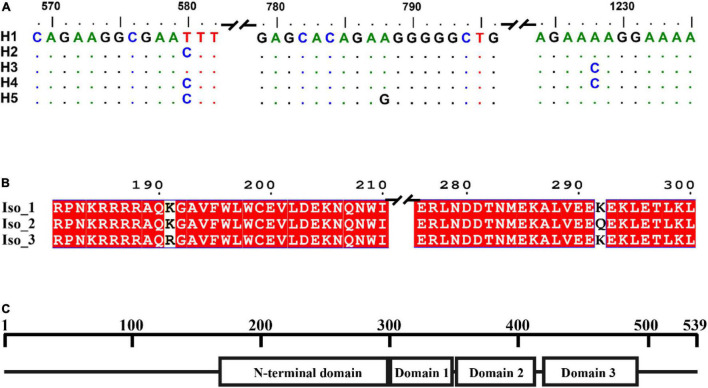
Gene and protein structure of *Phytophthora infestans* RAD4. **(A)** Nucleotide sequences including introns of the five haplotypes detected. Dots indicate identical nucleotides to the reference sequence, defined as Haplotype 1 (H1). **(B)** Deduced amino acid sequences in the isoforms (Iso). Different amino acids in the sequences were shown in white and shared amino acids were showed in red. **(C)** Protein structure predicted by IUPred2A using the reference sequence downloaded from NCBI (assessment number: 9474425).

A total of 140 full *RAD4* sequences were generated from the seven *P. infestans* populations. The *RAD4* gene was 1,974 nucleotides in length, containing four introns (nucleotides 85–225, 522–596, 797–892, and 1,160–1,201) and 1,620 bp coding sequence (CDS) regions which were translated to a protein with 539 amino acids. The 13.9% of total length amino acid residues in the protein are disordered. IUPred2A predicted that the protein was consisted of four domains (Figure 1C). Point mutations were the only mechanism generating genetic variation of *RAD4* gene sequences (Figure 1A). Two of the mutations (nucleotides 788 and 1,228) were found in CDS region whereas another one was found in nucleotide 580 in the intron region, generating a total of five haplotypes. The transition mutation from T to C in the nucleotide 580 of the intron changed Haplotype 1 (H1), the most common haplotype identical to the reference (assessment number: 9474425) downloaded from NCBI, to Haplotype 2 (H2) and a transversion mutation (A–C) in nucleotide 1,228 converted it to Haplotype 3 (H3). Haplotype 4 (H4) was generated from H1 by the combination of the two mutations documented in H2 and H3 whereas Haplotype 5 (H5) was generated from H1 by a mutations documented in H2 and a transition mutation (A–G) in nucleotide 788 (Figure 1A).

The average nucleotide identities in the full *RAD4* sequences among the 140 *P. infestans* isolates were 99.9%. Mean A, T, C, and G content in the CDS was 24.99, 21.54, 22.66, and 30.80%, respectively, and GC content was significantly higher than AT content (λ^2^ = 7.77, DF = 1, *p* = 0.0053). On the other hand, mean A, T, C, and G content in the introns was 29.66, 16.93, 23.75, and 29.66%, respectively, and GC content was not different to AT content (λ^2^ = 1.65, DF = 1, *p* = 0.1990).

**FIGURE 2:**
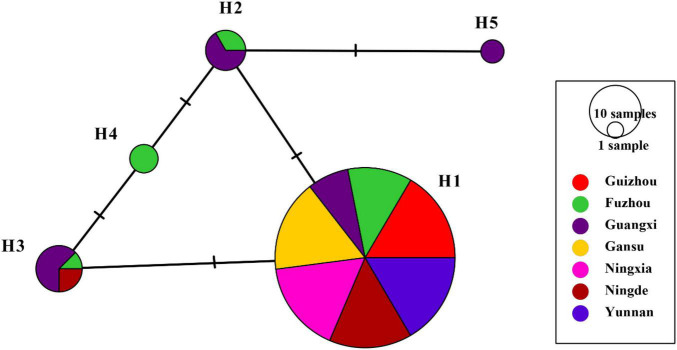
Nucleotide haplotype network of *RAD4* gene in the *Phytophthora infestans* populations sampled from seven production area in China. Each circle represents a unique haplotype, and the size of circles represents the proportion of the haplotypes in the sample pooled from the seven locations. The short-term marks represent steps of nucleotide substitution between two haplotypes.

H1 accounted for 86.43% of nucleotide sequences and was the main haplotype in all seven populations (Table 1). Only a single haplotype (H1) was recovered in Guizhou, Gansu, Ningxia, and Yunnan, the regions located in high altitudes and temperate-continental climatic zones with large area of potato production. All other haplotypes were found in Fujian (Fuzhou and Ningde) and Guangxi, the small potato production areas located in low altitude, subtropical climatic zones of Southern China. A total of four haplotypes each were detected in Fuzhou (H1, H2, H3, and H4) and Guangxi (H1, H2, H3, and H5) population and two haplotypes (H1 and H3) were found in Ningde population. The five haplotypes diverged by 1–3 mutation steps, and four of the haplotypes (H1, H2, H3, and H4) formed a reticulation structure in the haplotype network (Figure 2). Further analysis revealed a significant difference in haplotype frequency among populations from different locations (λ^2^ = 71.9, DF = 24, *p* < 0.0001).

**TABLE 1:** Frequency distribution of *RAD4* nucleotide haplotypes and isoforms in the seven *Phytophthora infestans* populations sampled from different altitudes (in parenthesis) of China.

Isoforms of amino acid	Haplotypes of nucleotide	Locations						
		Guizhou	Fuzhou	Guangxi	Gansu	Ningxia	Ningde	Yunnan
Iso_1	H1	1.00	0.70	0.45	1.00	1.00	0.90	1.00
	H2	0.00	0.10	0.20	0.00	0.00	0.00	0.00
Iso_2	H3	0.00	0.05	0.25	0.00	0.00	0.10	0.00
	H4	0.00	0.15	0.00	0.00	0.00	0.00	0.00
Iso_3	H5	0.00	0.00	0.10	0.00	0.00	0.00	0.00
Total		1.00	1.00	1.00	1.00	1.00	1.00	1.00

### Genetic Variation in the Deduced Isoform of *Phytophthora infestans RAD4*

The five haplotypes were translated into three isoforms among which only one amino acid differed from each other (Figure 1B and Table 1). H1 and H2 were deduced into the same isoform, i.e., Iso_1. H3 and H4 were also deduced into the same isoform, defined as Iso_2, due to the non-synonymous mutation at nucleotide 1,228, which changes lysine to glutamine acid at amino acid 292. The non-synonymous substitution from A in H1 to G in H5 at nucleotide 788th changed lysine acid to arginine at amino acid 191, creating another isoform, defined as Iso_3. The isoforms share the positions of three beta-hairpin domain (BHD1, BHD2, and BHD3), but were slightly different in the position and length of *RAD4* N-terminal domain (Table 2). The domain in Iso_1 was one amino acid shorter than that in Iso_2 but one amino acid longer than that in Iso_3. Both Iso_2 and Iso_3 were rare and only observed in three populations sampled from lower altitude regions, i.e., Ningde, Guangxi, and Fuzhou. Like haplotypes, a spatial heterogeneity in isoform frequency was detected (λ^2^ = 50.7, DF = 18, *p* < 0.0001).

**TABLE 2:** IUPred2A prediction of domain structure and position in the three isoforms of *Phytophthora infestans RAD4*.

Isoform	Predicted position of *RAD4* domains			
	N-terminal domain*	BHD1	BHD2	BHD3
Iso_1	169–300	301–349	353–414	421–494
Iso_2	169–301	301–349	353–414	421–494
Iso_3	170–300	301–349	353–414	421–494

*Includes the transglutaminase-homology (TGD) domain.

### Biophysical and Biochemical Analysis of *RAD4* Isoforms

**FIGURE 3:**
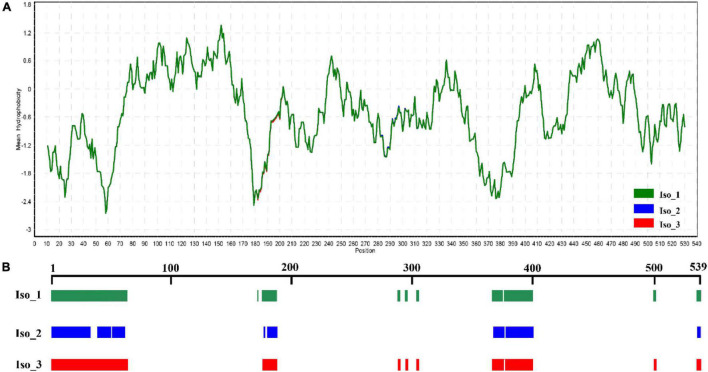
Hydrophilic estimate and structure prediction of Isoform-1: **(A)** Hydrophobicity estimated by a Kyte & Doolittle (K-D) approach embedded in the BioEdit program; and **(B)** intrinsically disordered protein regions (IDPRs) estimated by MobiDB.

**FIGURE 4:**
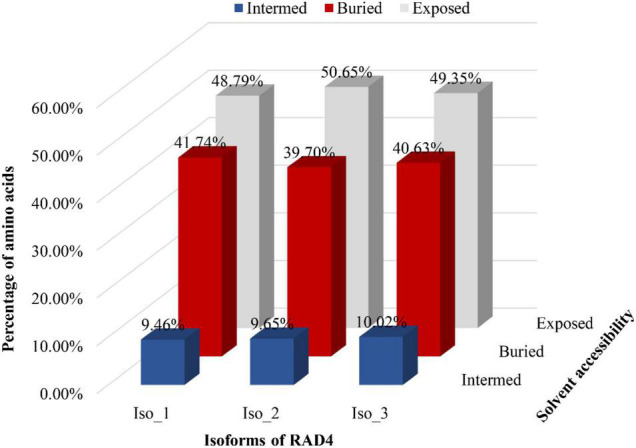
Solvent accessibility of three RAD4 isoforms. A system of neural networks with a window size of 17 was used to predict the solvent accessibility.

Radiation-sensitive 4 contained more hydrophilic amino acids than hydrophobic amino acids and possessed several intrinsically disordered protein regions (IDPRs, Figure 3). The level of hydrophobicity did not differ among the isoforms but Iso_1 had longer and more IDPRs than Iso_2 and Iso_3. Similarly, the mutations changed the secondary structure composition and solvent accessibility of the isoforms (Table 3 and Figure 4). Iso_1 had less loop but more helix than Iso_2 and Iso_3, corresponding to its less exposure to surface.

**TABLE 3:** Secondary structure of RAD4 isoforms predicted by Predict Protein.

Protein isoform	Helix*	Strand**	Loop
**Iso_1**	**32.65%**	**11.50%**	**55.84%**
Iso_2	30.61%	12.06%	57.33%
Iso_3	28.76%	12.06%	59.18%

*Includes alpha-, pi-, and 3_10-helix.

**Extended strand in beta-sheet conformation with two or residues.

***Bold values were the secondary structure about the main isoform of RAD4.

### Quantitative Real-Time PCR Analysis of *RAD4*

**FIGURE 5:**
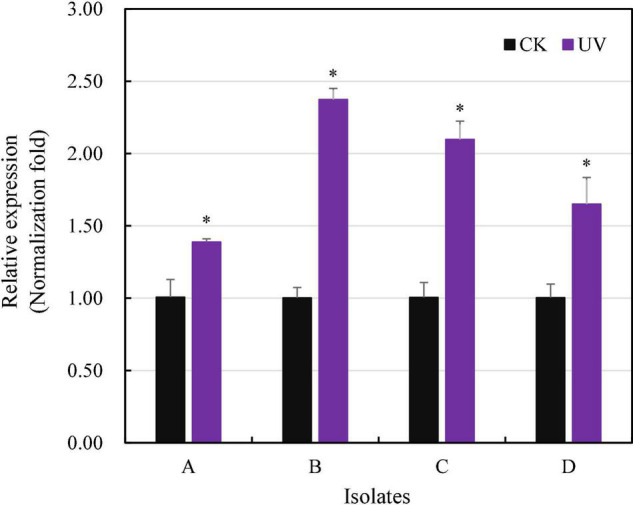
Quantitative real-time PCR analysis of *RAD4* expression. The qPCR was carried out by quantifying the expression of *RAD4* relative to the Actin A housekeeping gene using the 2-ΔΔCT method. The experimental isolates (A–D) were either exposed to 20-min UVC radiation in each of for 8-day period or without UV treatment (CK). ^∗^Indicates a significant difference (*p* < 0.05) between UV treated and non-treated isolates in *RAD4* expression.

Radiation-sensitive 4 transcription increased 1.39–2.37 times in the isolates treated by UV irradiation, and the increment was significant for all four experimental *P. infestans* isolates (*p* < 0.05, Figure 5). In addition, the level of *RAD4* upregulation also differed significantly among the isolates.

### Ultraviolet Tolerance of Three RAD4 Isoforms in *Phytophthora infestans*

Significant variation in colony growth rate and UV tolerance was found in the three isoforms. Iso_1 was the most tolerant to UV treatment whereas Iso_3 was the least (Table 4). The rank of UV tolerance and frequency in the three isoforms was well-matched even though limited data points prevented a meaningful analysis of regression association between the two parameters.

**TABLE 4:** Duncan’s multiple range test for difference in UVC tolerance among *RAD4* isoforms measured by the mycelia growth rate of *Phytophthora infestans* isolates treated with UVC relative to no UV treatment (RGR).

Isoform	*N* *	Mean RGR
Iso_1	2,587	0.94 ± 0.11 A**
Iso_2	210	0.91 ± 0.12 B
Iso_3	42	0.89 ± 0.07 B

*Number of data points included in the analysis. The data points were generated from 140 isolates in seven UVC treatments each with three replicates.

**Value in a column followed by different letters indicates statistical difference at p = 0.05.

## Discussion

Ultraviolet adaptation is a complex process that may be cooperatively regulated by many genes in the genome. It has been documented that RAD4 in several species is homologous to the mammalian NER protein xeroderma pigmentosum complementation group C (XPC) and is one of the genes contributing to UV adaptation (Lahari et al., 2018). Here, we confirm that *RAD4* also involves in UV adaptation of the pathogen *P. infestans*. This conclusion is synthesized from the observations of fitness polymorphisms and expression profiles of the *RAD4* gene. Significant change in UV tolerance was detected among the RAD4 isoforms altered by a single non-synonymous mutation, a pattern not observed in other studies, and the variations in UV adaptation and field frequency of the isoforms were concordant (Tables 1, 4). Species are equipped with an array of mechanisms to regulate their metabolic activities. For cost efficiency, many genes are expressed only when they are needed in survival and reproduction (Rivera et al., 2021). Our early study showed that physiological plasticity associated with gene expression contributed remarkably to UV adaptation of *P. infestans* (Wu et al., 2019). Consistent with these theories and observations, we found that expression of the *RAD4* gene was significantly upregulated when the pathogen was challenged by UV lights (Figure 5). Evaluation of the amino acid composition in the RAD4 protein further supports the involvement of the *RAD4* gene in UV adaptation of *P. infestans.* Leucine-rich repeats are important in DNA repair proteins (Nair et al., 2014). Likewise, leucine is the most frequent in the *RAD4* protein of *P. infestans*, which accounts for 12.62% (data not shown) of the structure.

Evolutionary strategies differ largely between conserved genes and other variant genes such as pathogenicity and tissue-specific genes (Alrefai et al., 2007). For example, many housekeeping genes are conserved in evolution due to their essential roles in providing fundamental service to the survival of a cell (Zhang and Li, 2004), whereas many effector genes of pathogens can quickly evolve in responding to host changes (Waheed et al., 2021). Low genetic variation was found in the *RAD4* gene studied. Only three point mutations were detected in the 140 *RAD4* full-length sequences (Figure 1A), similar to other conserved genes such as *RAD23* and housekeeping *eEF-1*α characterized from *P. infestans* (Wang et al., 2020, 2021).

Different parts of genomes in the same species can dramatically vary in mutation and recombination rates (Stukenbrock and Croll, 2014). Conserved evolution in *RAD4* of *P. infestans* could be due to its low mutation and recombination rate which limit the generation of genetic variation. However, it is worthy of note that a significant higher GC (53.46%) than AT (46.53%) content was found in the 140 *RAD4* CDS sequences. High GC content favors for methylation, which is prone to mutation (Pfeifer, 2006). Mutation rates in introns are usually similar across different parts of a genome (Hare and Palumbi, 2003). Among the three mutations observed, two were in exons which have 1,622 nucleotides in total, and another was in introns which have 352 nucleotides in total. The ratio of mutation site to nucleotide number is not different between the two parts of genome (λ^2^ = 0.47, DF = 1, *p* = 0.4943), falsifying the hypothesis of low mutation rate in the coding regions of *RAD4* gene. Furthermore, considering the reticulation structure in haplotype network, recombination within the gene might not be rare.

Even though low mutation cannot be completely excluded, we believe that purifying selection is the main genetic force responsible for the conserved evolution in *RAD4* sequences, and we have several lines of evidence to support the hypothesis, aligning with theories and empirical analyses of many conserved genes (Jackson et al., 2015). First, non-random spatial heterogeneity was found among the deduced isoforms (Table 1). All isolates from higher altitudes belong to Iso_1, the likely ancestry isoform whereas its descanting isoforms only exist in low altitudes. Our previous results also found a gradient distribution along altitudes in UV tolerance and sequence characters of *RAD23* gene in the pathogen (Wang et al., 2021). Second, Iso_1, the ancestry isoform, outperformed the descants on average under UV stress, suggesting its overall higher fitness. This result is consistent with the expectation that mutations in conserved genes can compromise their fitness and many of the mutants would be purged out to reduce variation (Zhang and Li, 2004). Third, there was difference in GC/AT between introns and exons. In introns, the percentage of GC and AT bases was almost identical, consistent with neutral expectation in this part of genomes (Hildebrand et al., 2010). On the other hand, GC bases were overrepresented in exons of the gene.

Like in other species (Min and Pavletich, 2007), four domains were predicted from the RAD4 protein of *P. infestans*. Among them, the N-terminal domain including TGD is the least conserved. It houses all non-synonymous mutations detected in the study and varies both in length and in position (Table 2). Even though a single amino acid substitution can cause 3–5% fitness deduction (Table 4) in terms of UV adaptation, this result indicates that the N-terminal domain can tolerate more mutations than other domains in RAD4. This variation in sequence resilience of the domains may reflect their functional difference in NER system. During DNA repairing, TGD binds to the healthy template DNA and therefore does not involve in the recognition of damaged lesions as other domains do (Velmurugu et al., 2016).

Amino acid substitutions by non-synonymous mutations change three-dimensional structure and properties of proteins and therefore affect binding affinity of proteins with other molecules (Gress et al., 2017). Bioinformatic prediction found remarkable variation in the secondary structure and solvent accessibility among the three RAD4 isoforms (Figure 4 and Table 3). The mutations in Iso_1 reduce protein helix content but increase loop content of the RAD4 protein, leading to 5 and 11% less helix but 3 and 6% more loop in Iso_2 and Iso_3, respectively. Protein helix plays an important role in binding with substrates whereas loop determines the stability of proteins (Lehmann and Bass, 1999). Consequently, protein with high helix tends to bind better with substrates and those with high loop tend to be less stable. In transporting system of human, helix motif regulates the binding affinity of apolipoprotein A-I with lipids (Saito et al., 2004). In NER system, helixes in *RAD4* (Gietz and Prakash, 1988) and other proteins (Massari and Murre, 2000) are also required to form the functional repairing apparatus. The progressive decrease of helix but increase of loop from Iso_1 to Iso_2 and Iso_3 may contribute the corresponding reduction of UV tolerance in the three isoforms.

The recent crystal studies and bioinformatic inferences reveal that IDPRs play an important role in facilitating molecular recognition and binding with receptors to form protein complexes because of their structural flexibility. These unique structures involve in many biological and ecological activities such as species adaptation to the stresses induced by host immunity and abiotic environments including UV lights and temperature (Wang et al., 2008; Liu et al., 2013). For example, single amino acid substitution in an avirulent *P. infestans* effector generates several IDPRs, which covert the avirulence effector to a virulence effector which is able to escape immunity recognition (Yang et al., 2020). Here, we also found that protein ordering status in RAD4 may contribute to UV adaptation of *P. infestans.* The non-synonymous mutations in the *RAD4* gene did not change protein hydrophobicity but substantially affect the size of IDPRs (Figures 3, 4). The mutants, particularly Iso_2, have much less IDPRs than Iso_1 (Figure 3B). Correspondingly, Iso_1 displays significantly higher UV tolerance than both Iso_2 and Iso_3 (Table 4).

Taken together, we found that *RAD4* is an important gene regulating UV adaptation in *Phytophthora infestans*, and a single point mutation of this gene can significantly alter the fitness of the pathogen. Our results are derived from population genetics and fitness comparisons of multiple mutants in natural populations. The advantage of this approach is that it can include pathogen individuals of many genetic backgrounds and avoid invasive experiments by artificially altering the genome structure of the species, which is not feasible in many cases due to ethical considerations (e.g., humans) or technical limitations. Although GMOs are routinely adopted nowadays, they have proven very difficult for some species, such as *Phytophthora infestans*, and are therefore not a research focus in our laboratory. However, as one reviewer pointed out, generating a set of transgenic *Phytophthora infestans* strains with different mutants followed by fitness testing is the better choice for direct confirmation of RAD4 function and should be attempted in the future.

## Data Availability Statement

The original contributions presented in this study are included in the article/Supplementary Material, further inquiries can be directed to the corresponding authors.

## Author Contributions

JZ conceived and designed the experiments. Y-PW and L-NY collected the *P. infestans* isolates. Y-PW, L-NY, and Y-YF performed the experiments and generated the data. Y-PW analyzed and interpreted the data. JZ and SL supervised the project. Y-PW and JZ wrote the manuscript. All authors contributed to the article and approved the submitted version.

## Conflict of Interest

The authors declare that the research was conducted in the absence of any commercial or financial relationships that could be construed as a potential conflict of interest.

## Publisher’s Note

All claims expressed in this article are solely those of the authors and do not necessarily represent those of their affiliated organizations, or those of the publisher, the editors and the reviewers. Any product that may be evaluated in this article, or claim that may be made by its manufacturer, is not guaranteed or endorsed by the publisher.

## Footnotes

1
http://technelysium.com.au/wp/

2
http://espript.ibcp.fr/ESPript/ESPript/

3
http://www.predictprotein.org

4
https://iupred2a.elte.hu/

5
https://mobidb.bio.unipd.it/
