# Corrigendum: Single amino acid substitution in the DNA repairing gene *radiation-sensitive 4* contributes to ultraviolet tolerance of a plant pathogen

**DOI:** 10.3389/fmicb.2022.1005752

**Published:** 2022-08-15

**Authors:** Yan-Ping Wang, Li-Na Yang, Yuan-Yuan Feng, Songqing Liu, Jiasui Zhan

**Affiliations:** ^1^Sichuan Provincial Key Laboratory for Development and Utilization of Characteristic Horticultural Biological Resources, College of Chemistry and Life Sciences, Chengdu Normal University, Chengdu, China; ^2^Institute of Oceanography, Minjiang University, Fuzhou, China; ^3^Department of Forest Mycology and Plant Pathology, Swedish University of Agricultural Sciences, Uppsala, Sweden

**Keywords:** nucleotide excision repair system, climate change, UV adaptation, population genetics, natural selection, transcriptional regulation, evolutionary ecology, agriculture

In the published article, there was an error made in the article title and the missed of one meaningful preposition “in” in the title.

Instead of “Single amino acid substitution the DNA repairing gene *radiation-sensitive 4* contributes to ultraviolet tolerance of a plant pathogen,” it should be “Single amino acid substitution in the DNA repairing gene *radiation-sensitive 4* contributes to ultraviolet tolerance of a plant pathogen.”

The authors apologize for this error and state that this does not change the scientific conclusions of the article in any way. The original article has been updated.

## Publisher's note

All claims expressed in this article are solely those of the authors and do not necessarily represent those of their affiliated organizations, or those of the publisher, the editors and the reviewers. Any product that may be evaluated in this article, or claim that may be made by its manufacturer, is not guaranteed or endorsed by the publisher.

