# Supplementary material for: Single Amino Acid Substitution in the DNA Repairing Gene Radiation-Sensitive 4 Contributes to Ultraviolet Tolerance of a Plant Pathogen
Source: Front Microbiol. 2022 Jul 14;13:927139. doi: 10.3389/fmicb.2022.927139 (PMC9330021; doi:10.3389/fmicb.2022.927139)
Supplement: Supplementary file 1 [file Table_1.pdf]

Table S1. Altitude, sample size and *RAD4* haplotypes and isoforms of the seven *Phytophthora infestans* populations were collected

Populations	Altitude (m)	Sample size	RAD4 haplotypes	RAD4 isoforms
Guizhou	133	20	H1	Iso_1
Fuzhou	10	20	H1, H2, H3, H4	Iso_1, Iso_2
Guangxi	78	20	H1, H2, H3, H5	Iso_1, Iso_2, Iso_3
Gansu	2088	20	H1	Iso_1
Ningxia	1778	20	H1	Iso_1
Ningde	31	20	H1, H3	Iso_1, Iso_2
Yunnan	2676	20	H1	Iso_1

Table S2. Information of primer sequences

Primer name	Primer sequence	Amplification length (bp)	Annealing temperature (℃)	Extension time (s)
RAD4-F1	AGCGGAATAAAGCTGGTTGAG	741	56	45
RAD4-R1	GCTCGTAGTCTTGTCCTTCTC			
RAD4-F2	GCAACTCGCATCAAATGGACTCAAC	1089	55	65
RAD4-R2	ACGCTCGCTCTCCTTCACTTCT			
RAD4-F3	TCTGGAAGGATTCAGGAAGCAT	829	55	50
RAD4-R3	AACACGGTCCAAACGATGAAAT			
